# New Steroidal Glycosides Isolated as CD40L Inhibitors of Activated Platelets

**DOI:** 10.3390/molecules15074589

**Published:** 2010-06-25

**Authors:** Haifeng Chen, Wenchao Ou, Guanghui Wang, Naili Wang, Linnan Zhang, Xinsheng Yao

**Affiliations:** 1 Institute for Biomedical Research, Xiamen University, Xiamen 361005, China; E-Mails: haifeng@xmu.edu.cn (H.-F.C.); guanghui@xmu.edu.cn (G.-H.W.); 2 Department of Cardiology, The Second Affiliated Hospital, Guangzhou Medical University, Guangzhou 510260, China; 3 Department of Natural Products Chemistry, Shenyang Pharmaceutical University, Shenyang 110016, China; E-Mail: wangnl@sz.tsinghua.edu.cn (N.-L.W.); 4 Clinical Medicine & Pharmacy College, China Medical University, Shenyang 110001, China; E-Mail: z.lnn@163.com (L.-N.Z.)

**Keywords:** steroidal glycosides, *Allium macrostemon* Bunge, CD40L inhibitors

## Abstract

Three new compounds were isolated from the dried bulbs of *Allium macrostemon* Bunge. Their structures were elucidated from their spectral data as (25*R*)-26-*O*-*β*-D-glucopyranosyl-5*α*-furostane-3*β*,12*β*,22,26-tetraol-3-*O*-*β*-D-glucopyranos-yl (1→2) [*β*-D-glucopyranosyl (1→3)]-*β*-D-glucopyranosyl (1→4)-*β*-D-galactopyranoside (**1**), (25*R*)-26-*O*-*β*-D-glucopyranosyl-5*α*-furostane-3*β*,12*α*,22,26-tetraol-3-*O*-*β*-D-gluco- pyranosyl (1→2) [*β*-D-glucopyranosyl (1→3)]-*β*-D-glucopyranosyl (1→4)-*β*-D-galacto- pyranoside (**2**) and (25*R*)-26-*O*-*β*-D-glucopyranosyl-5*β*-furostane-3*β*,12*α*,22,26-tetraol-3-*O*-*β*-D-glucopyranosyl (1→2)-*β*-D-galactopyranoside (**3**), respectively. The inhibition effect of all compounds on CD40 ligand (CD40L) expression on the membrane of activated platelets stimulated by ADP was tested. Compounds **1** and **2** exhibited significant inhibitory activities in a dose dependent manner (P < 0.05), suggesting their potential application as CD40L inhibitors.

## 1. Introduction

Recent reports suggest that most anti-platelet agents can be used as anti-inflammatory drugs and platelet activation is sometimes critical in the development of inflammation [1]. As a member of the tumor necrosis factor-α family of proteins, CD40L has been identified as a proinflammatory mediator and risk factor for cardiovascular events on activated platelets [2]. It can bind and activate platelet *α*_Ⅱb_*β*_3_ in thrombosis and inflammation [3,4]. It is also able to activate endothelial cells to have a proinflammatory phenotype [5]. The stimulation of endothelial cells by platelets expressing CD40L contributes to inflammatory cell recruitment in atherosclerosis [6]. And the investigation on CD40L inhibitor would be significant and useful.

It was reported that, as the main constituents of dried bulbs of *Allium macorstemon* Bunge, steroidal glycosides could strongly inhibit the platelet aggregation of human beings [7]. But their activity on platelet inflammatory was never reported. This paper concerns the isolation and structure elucidation of three new steroidal glycosides from ethanol extract of the dried bulbs of *Allium macorstemon* Bunge. Their inhibition on CD40L expression on the membrane of activated platelets was tested. 

## 2. Results and Discussion

Compound **1** was isolated as an amorphous powder. The molecular formula was determined as C_57_H_96_O_30_ by the HR-ESIMS peak at *m/z* 1283.5862 [M+Na]^+^. The positive reaction to the Ehrlich reagent suggests a furostanol glycoside structure for **1** [8]. The ^1^H-NMR spectrum of **1** revealed the presence of four methyl groups at *δ* 1.34 (s, Me-18), 0.66 (s, Me-19), 1.60 (d, *J* = 6.8 Hz, Me-21) and 0.97 (d, *J* = 6.8 Hz, Me-27) (Table 1). 

**Table 1:** ^1^H-NMR data of **1**, **2** and **3** (C_5_D_5_N, *δ* ppm) ^a^.

No.	1	2	3	No.	1	2	3
1	0.78,1.51 (o)	0.82, 1.51 (o)	1.51,1.80 (o)	Inner Gal-1	4.85(d, *J* = 7.8 Hz)	4.84(d, *J* = 7.8 Hz)	4.84(d, *J* = 7.6 Hz)
2	1.24,1.99 (o)	1.25, 1.30 (o)	1.07,1.64 (o)	2	4.42(o)	4.43(o)	4.63(o)
3	4.02(o)	3.80 (o)	4.28 (o)	3	4.19(o)	4.20(o)	4.20 (o)
4	1.31,1.77 (o)	1.35, 1.75 (o)	1.51,1.80, (o)	4	4.58(m)	4.56(m)	4.54 (o)
5	0.84(o)	0.92 (o)	2.19 (o)	5	4.16(o)	4.14(o)	3.96 (o)
6	1.08,1.11 (o)	1.10, 1.60 (o)	1.19,1.87 (o)	6	4.21,4.68(o)	4.67,4.21(o)	4.39, 4.43(o)
7	0.47,1.55 (o)	1.57, 1.99 (o)	1.31,1.89 (o)	Gal-Glc-1	5.56(d, *J* = 7.8 Hz)	5.56(d, *J* = 7.8 Hz)	5.25(d, *J* = 7.6 Hz)
8	1.43(o)	1.57 (o)	1.66 (o)	2	4.38(o)	4.38(o)	4.06 (o)
9	0.65(o)	1.33 (o)	2.11 (o)	3	4.19(o)	4.17(o)	4.16 (o)
10	----	------	----	4	3.84(o)	3.86(o)	4.29 (o)
11	1.49,1.82 (o)	1.63, 1.78 (o)	1.63,1.72 (o)	5	4.13(o)	4.13(o)	3.93 (o)
12	3.52(m)	3.98 (o)	4.21 (o)	6	4.51(o)	4.50(o)	4.38, 4.43 (o)
13	----	------	----	3-Glc-1	5.28(d, *J* = 7.8 Hz)	5.29(d, *J* = 7.8 Hz)	
14	1.13(o)	2.06 (o)	2.12 (o)	2	4.02(o)	4.00(o)	
15	1.52,2.08 (o)	0.89 (o)	1.53,2.13 (o)	3	3.84(o)	3.85(o)	
16	5.02(o)	5.02 (o)	5.04 (m)	4	4.23(o)	4.23(o)	
17	2.31(t, *J* = 8.2 Hz)	3.21 (dd, *J* = 7.2, 8.3 Hz)	3.22 (dd, *J* = 6.8, 8.7 Hz)	5	3.85(o)	3.83(o)	
18	1.34 (s)	0.99 (s)	0.99 (s)	6	4.18,4.27(o)	4.18,4.27(o)	
19	0.66(s)	0.68 (s)	1.02 (s)	2-Glc-1	4.77(d, *J* = 7.8 Hz)	4.76(d, *J* = 7.8 Hz)	
20	2.48(m)	2.28 (o)	2.29 (m)	2	4.08(o)	4.09(o)	
21	1.60(d, *J* = 6.8 Hz)	1.38(d, *J* = 6.8 Hz)	1.36(d, *J* = 6.8 Hz)	3	4.21(o)	4.21(o)	
22	----	------	----	4	4.17(o)	4.18(o)	
23	2.08(o)	2.10 (o)	2.04, 2.06 (o)	5	3.92(o)	3.92(o)	
24	1.69,2.08 (o)	1.70, 2.08 (o)	1.71, 2.06 (o)	6	4.20,4.00(o)	4.21,4.00(o)	
25	1.91(o)	1.93 (o)	1.94 (o)	C_26_ Glc-1	5.13(d, *J* = 7.8 Hz)	5.12(d, *J* = 7.8 Hz)	4.80(d, *J* = 7.6 Hz)
26	3.61(m) 3.90(o)	3.62 (o)	3.63 (o)	2	4.00(o)	4.01(o)	4.22 (o)
		3.99 (m)	4.00 (m)				
27	0.97(d, *J* = 6.8 Hz)	0.99(d, *J* = 6.8 Hz)	0.99(d, *J* = 6.8 Hz)	3	4.03(o)	4.04(o)	3.83 (o)
				4	4.23(o)	4.23(o)	3.99 (o)
				5	4.17(o)	4.16(o)	4.22 (o)
				6	4.20,4.49(o)	4.20,4.47 (o)	4.37,4.52 (o)

^a^ Recorded on a Bruker-400 NMR spectrometer.

The anomeric region of the ^1^H-NMR spectrum showed five anomeric proton signals at δ_H_ 4.77 (d, *J* = 7.8 Hz), 4.85 (d, *J* = 7.8 Hz), 5.13 (d, *J* = 7.8 Hz), 5.28 (d, *J* = 7.8 Hz) and 5.56 (d, *J* = 7.8 Hz), respectively. Large coupling constants (*^3^J_1, 2_* = 7.8 Hz) for anomeric protons revealed the *β-*configuration of all sugars. The ^13^C-NMR and DEPT spectra showed 57 carbon signals, including four methyls, 35 methylenes, 15 methines and three quaternary carbons (Table 2). 

**Table 2:** ^13^C-NMR data of **1**, **2** and **3** (C_5_D_5_N, *δ* ppm)^a^.

No.	1	2	3	No.	1	2	3
1	37.1	37.0	30.8	Inner Gal-1	102.4	102.3	102.3
2	29.8	29.9	26.8	2	73.1	73.1	81.7
3	75.1	77.5	75.1	3	75.5	75.5	75.1
4	34.7	34.8	30.9	4	80.1	80.2	69.8
5	44.7	44.8	37.0	5	76.1	76.1	76.5
6	28.9	29.0	27.1	6	60.5	60.5	62.1
7	32.2	29.8	26.7	Gal-Glc-1	104.8	104.9	106.0
8	34.3	35.6	35.9	2	81.4	81.4	76.8
9	53.5	47.8	33.7	3	88.4	88.4	78.0
10	35.8	35.5	34.9	4	70.8	70.8	71.7
11	31.6	29.5	29.6	5	77.8	77.8	78.4
12	79.3	71.5	71.7	6	62.3	62.3	62.8
13	46.8	45.6	45.8	3-Glc-1	104.5	104.5	
14	55.0	48.3	48.6	2	75.1	75.1	
15	32.1	32.5	32.5	3	78.6	78.6	
16	81.1	80.9	81.0	4	70.9	70.9	
17	63.7	54.5	54.6	5	77.5	77.4	
18	11.3	17.4	17.5	6	62.7	62.3	
19	12.2	12.2	23.9	2-Glc-1	104.8	104.8	
20	41.6	40.9	40.9	2	75.1	75.2	
21	15.6	16.2	16.2	3	78.6	78.6	
22	110.8	110.6	110.7	4	71.7	71.7	
23	37.3	37.2	37.2	5	78.4	78.6	
24	28.4	28.4	28.4	6	62.8	62.8	
25	34.2	34.2	34.3	C_26_ Glc-1	105.0	105.0	104.9
26	75.2	75.3	75.3	2	75.2	75.3	75.1
27	17.4	17.4	17.4	3	78.6	78.6	78.3
				4	71.5	71.5	71.6
				5	78.5	78.4	78.5
				6	63.0	63.0	62.8

^a^ Recorded on a Bruker-400 (100 MHz for ^13^C) NMR spectrometer.

**Figure 1:**
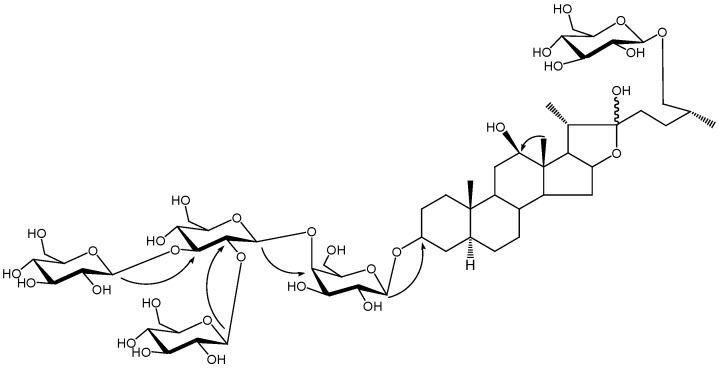
Key HMBC correlations of compound **1.**

Comparison of the ^13^C-NMR data of **1 **and those of the reported compound ((25*R*) 26-*O*-*β* -D-glucopyranosyl-3*β* ,22 ,26-trihydroxyl-5afurostane-3-*O*-*β* -chacotrioside)[8] suggested a considerable structural similarity except for the carbon signals around C-12. Comparison of the aglycone to the reported compound indicated the existence of a *β*-hydroxyl group at C-12, which led to the similar ^13^C-NMR shifts of the carbons C-11, C-12 and C-13 [9,10]. This was also supported by HMBC correlations (Figure 1) between the proton signal at δ_H_ 1.34 (H-18) and carbon signal at δ_C_ 79.3 (C-12). The 25*R* configuration was determined by the chemical shifts of C-23 (δ_C_ 37.3), C-24 (δ_C_ 28.4), C-25 (δ_C_ 34.2), C-26 (δ_C_ 75.2) and C-27 (δ_C_17.4) [11,12]. This was also confirmed by the difference between the chemical shifts of two proton signals of H-26 (0.29) since the difference is usually >0.57 for (25*S*) configurations and <0.48 for (25*R*) configurations [13]. The chemical shifts of C-5 (δ_C_ 44.7), C-9 (δ_C_ 53.5) and C-19 (δ_C_ 12.2) were consistent with those of the 5*α*-steriodal glycosides [8,10,14], indicating the H-5*α* configuration.

**Figure 2:**
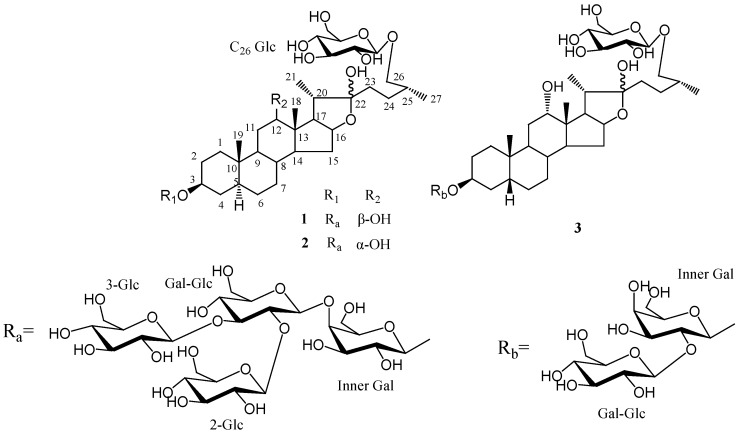
The structures of compounds **1**–**3**.

Acid hydrolysis of **1** yielded D-glucose and D-galactose in a ratio of 4:1. The HMBC correlations from δ_H_ 4.85 (H-1 of inner galactose attached to C-3 of the aglycone) to δ_C_ 75.1 (C-3 of aglycone), from δ_H_ 5.56 (H-1of inner glucose) to δ_C_ 80.1 (C-4 of inner galactose), from δ_H_ 5.28 (H-1of terminal glucose attached to inner glucose) to δ_C_ 88.4 (C-3 of inner glucose), from δ_H_ 4.77 (H-1of terminal glucose attached to inner glucose) to δ_C_ 81.4 (C-2 of inner glucose) and from δ_H_ 5.13 (H-1 of terminal glucose attached to C-26) to δ_C_ 75.2 (C-26 of aglycone) indicated that two sugar chains were attached to C-3 and C-26 of the aglycone, respectively. A terminal glucose was attached at C-26. The inner glucose was linked at C-4 of the inner galactose attached to C-3 and two terminal glucoses were linked at C-3 and C-2 of the inner glucose. The combined use of ^1^H-^1^H COSY, HSQC, TOCSY and HMBC experiments allowed the sequential assignments of all resonances for each monosaccharide. Compound **1** was thus identified as (25*R*)-26-*O*-*β*-D-glucopyranosyl-5*α*-furostane-3*β*,12*β*,22,26-tetraol-3-*O*-*β*-D-gluco- pyranosyl(1→2) [*β*-D-glucopyranosyl (1→3)]-*β*-D-glucopyranosyl (1→4)-*β*-D-galactopyranoside (Figure 2).

Compound **2 **was isolated as a white amorphous powder. The same molecular formula of C_57_H_96_O_30_as **1** was deduced by the positive-ion HR-ESIMS signal at *m/z* 1283.5891 [M+ Na]^+^. Comparison of ^1^H-NMR and ^13^C-NMR spectra for the sugar moieties of **2** with those of **1** suggested the same sugar chains. The ^13^C-NMR data for the aglycone of **2** showed considerable similarity to **1** except for the signals around C-12. The long rang correlations of HMBC between methyl proton signal at δ_H_ 0.99 (H-18) and carbon signal at δ_C_ 71.5, proton signal at δ_H_ 3.98 (H-12) and carbon signal at δ_C_ 29.5 (C-11) and 45.6 (C-13) revealed the presence of hydroxyl group at C-12. The NOESY correlation between δ_H_ 3.98 (H-12) and δ_H_ 0.99 (H-18) suggested its *α*-orientation. The presence and configuration of C-12 hydroxyl group was also supported by carbon signals of C-11, C-12, C-13, C-14 and C-17 of **2 **shifted higher field by approximately – 2.1, - 7.8, - 1.2, - 6.7 and - 9.2 ppm, respectively, while the carbon signal of C-18 shifted to down field by +6.1 ppm comparing with those of **1**. Therefore, the structure of **2** was established as (25*R*)-26-*O*-*β*-D-glucopyranosyl-5*α*-furostane-3*β*,12*α*,22,26-tetraol-3-*O*-*β*-D-gluco- pyranosyl (1→2) [*β*-D-glucopyranosyl (1→3)]-*β*-D-glucopyranosyl (1→4)-*β*-D-galactopyranoside (Figure 2). 

Compound **3** was obtained as a white amorphous powder. The molecular formula of **3** was defined as C_45_H_76_O_20_ on the basis of the HRESI-MS signal at *m/z* 937.5075 [M+H]^+^. ^1^H-NMR and ^13^C-NMR data for the aglycone moieties of **3** was similar to those of **2**, except for the signals of A/B ring portion. In the ^13^C-NMR spectrum, the higher field chemical shift of the A/B ring of **3** and the downfield chemical shift of C-19 revealed the *β*-orientation ofH-5. This was also confirmed by NOESY correlation between proton signals at δ 2.19 (H-5) and 1.02 (H-19). The ^1^H-NMR and ^13^C-NMR spectra of **3** showed three anomeric protons at δ_H_ 4.80 (d, *J* = 7.6 Hz), 4.84 (d, *J* = 7.6 Hz) and 5.25 (d, *J* = 7.6 Hz) and three anomeric carbons at δ_C_ 104.9, 102.3 and 106.0, suggesting that there were only three sugar units in **3** and the same large constants (*^3^J_1,2_* = 7.6 Hz) for anomeric protons revealed the *β-*configuration of all these sugars. Acid hydrolysis of **3** yielded D-glucose and D-galactose in a ratio of 2:1. In the HMBC spectrum, long-range correlations from δ_H_ 4.84 (H-1 of inner galactose) to δ_C_ 75.1 (C-3 of aglycone), from δ_H_ 5.25 (H-1 of terminal glucose) to δ_C_ 81.7 (C-2 of inner galactose) and from δ_H_ 4.80 (H-1 of terminal glucose attached to C-26) to δ_C_ 75.3 (C-26 of aglycone) showed that two sugar chains were separately attached to C-3 and C-26 of the aglycone, and two glucoses were linked at C-2 of the inner galactose attached at C-3 and C-26 of aglycone respectively. Thus, the structure of **3 **was established as (25*R*)-26-*O*-*β*-D- glucopyranosyl-5*β*-furostane-3*β*,12*α*,22,26-tetraol-3-*O*-*β*-D-glucopyranosyl(1→2)-*β*-D-galactopyranoside (Figure 2).

**Figure 3:**
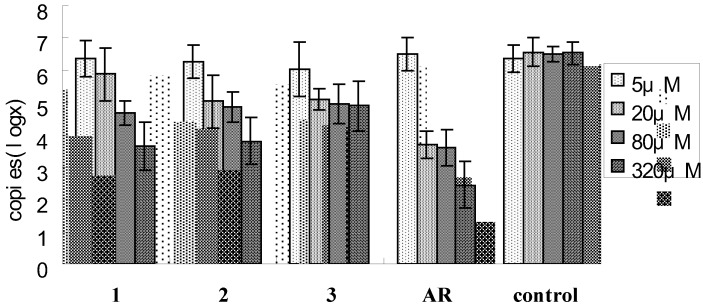
Inhibitory effect of the compounds **1**-**3** on CD40 ligand (CD40L) expression on the membrane of activated platelets stimulated by ADP.

The inhibitory effect of compounds **1**-**3** of CD40 ligand (CD40L) expression on the membrane of activated platelets stimulated by ADP was measured by real time PCR. Compounds **1** and **2** exhibited significantly inhibit expressions of CD40L in ADP induced active platelets in a dose dependent manner (P < 0.05) (Figure 3) which suggested that they may be new type of CD40L inhibitors for curing platelet inflammatory related diseases.

## 3. Experimental

### 3.1. General

Melting points were determined with a Yanaco MP-S_3_ micro-melting point apparatus and are uncorrected. Optical rotations were obtained on a P-1020 digital polarimeter (JASCO Corporation). IR spectra were measured on a Shimadzu FT/IR-8400 spectrometer. 1D and 2D NMR spectra were taken on a Bruker AV-400 (400 MHz for ^1^H-NMR) spectrometer in C_5_D_5_N solution. ESIMS spectra were acquired using a Bruker Esquire 2000 mass spectrometer. Column chromatography was carried out on Diaion HP-20 (Mitsubishi Kasei, Japan), silica gel (200–300 mesh, Qingdao Factory of Marine Chemical Industry, Qingdao, China) and ODS (40-63 μm, Merck). TLC analyses were taken on Silica gel 60F_254_ (Qingdao Factory of Marine Chemical Industry, Qingdao, China) and the spots were detected spraying with Ehrlich reagent and heating. Preparative HPLC was performed using an ODS column (250 × 20 mm, 10 μm, Shimadzu Pak; Detector: RID). Quantitative real-time PCR was performed using the PE5700 PCR system (Biosystems, Foster, CA, USA). The Qiagen RNeasy minikit system (Qiagen, Valencia, CA, USA) was used to collect mRNA from ADP induced active platelets. The mRNA was converted to cDNA using the AMV Reverse Transcription System (Promega, Madison, Wi, USA).

### 3.2. Plant material

The bulbs of *Allium Macrostemon* Bunge were purchased from Liaoning Province of China and identified by Professor Qi-Shi Sun (Department of Pharmacognosy, Shenyang Pharmaceutical University). The voucher specimen has been deposited at the Department of Natural Product Chemistry, Shenyang Pharmaceutical University, Shenyang (110016), P. R. China.

### 3.3. Extraction and isolation

The dried bulbs of *Allium Macrotemon* Bunge (5 kg) were extracted twice with 60% ethanol (10 L) for 2 h each time. The ethanol extraction was concentrated under reduced pressure, suspended in water and then passed through Diaion HP-20 column using EtOH/H_2_O gradient system (0–100%). The 60% EtOH eluted fraction (90 g), which was subjected to silica gel column chromatography with CHCl_3_/MeOH/H_2_O (9:1:0.1; 8:2:0.2; 7:3:0.5: 6:4:0.8, v/v/v) and MeOH finally, gave nine fractions. Fraction 2 was further purified by ODS column chromatography eluting with MeOH/H_2_O (3:7; 2:3; 1:0, v/v) to afford five sub fractions. The third subfraction was also further purified by repeated Rp-18 HPLC preparation, eluting with MeOH/H_2_O (50:50, v/v), to yield **1 **(8.1 mg), **2** (8.5 mg) and **3 **(30.6 mg). 

*Compound* **1*****(***(25*R*)-26-*O*-*β*-D-glucopyranosyl-5*α*-furostane-3*β*,12*β*,22,26-tetraol-3-*O*-*β*-D-gluco- pyranosyl(1→2) [*β*-D-glucopyranosyl (1→3)]-*β*-D-glucopyranosyl (1→4)-*β*-D-galactopyranoside)*.* White amorphous powder, m.p. 216–217 ºC, 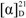-26.4° (H_2_O,*c* 0.11). HR-ESIMS (positive mode) at *m/z* 1283.5862 [M+ Na]^+^ (calcd. 1283.5884). ESI-MS (positive mode) at *m/z* 1283 [M+ Na]^+^, 1265 [M+ Na- H_2_O]^+^, 1121 [M+ Na- 162]^+^, 1103 [M+ Na- H_2_O- 162]^+^, 941 [M+ Na- H_2_O- 162× 2]^+^, 779 [M+ Na- H_2_O- 162× 3]^+^; ESI-MS (negative mode) at *m/z* 1259 [M- H]^-^, 1097 [M- H- 162]^-^, 935 [M- H- 162× 2]^-^, 773 [M- H- 162× 3]^-^, 611 [M- H- 162× 4]^-^; IR ν_max_ (KBr) cm^-1^: 3419 (OH), 2937 (CH), 1000-1100; ^1^H-NMR (C_5_D_5_N) data see Table 1; ^13^C-NMR, see Table 2.

*Compound **2 ******(***(25*R*)-26-*O*-*β*-D-glucopyranosyl-5*α*-furostane-3*β*,12*α*,22,26-tetraol-3-*O*-*β*-D-gluco- pyranosyl (1→2) [*β*-D-glucopyranosyl (1→3)]-*β*-D-glucopyranosyl (1→4)-*β*-D-galactopyranoside). White amorphous powder, m.p. 177–178 ºC, 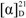-3.5° (H_2_O, *c* 0.16). HR-ESIMS (positive mode) at *m/z* 937.5075 [M+ H]^+^ (calcd. 937.5008). ESI-MS (positive mode) at *m/z* 959 [M+ Na]^+^, 941 [M+ Na- H_2_O]^+^, 919 [M+ H- H_2_O]^+^, 797 [M+ Na- 162]^+^, 617 [M+ Na- H_2_O- 162× 2]^+^, 595 [M+ H- H_2_O- 162× 2]^+^, 433 [M+ H- H_2_O- 162×2- 162]^+^; ESI-MS (negative mode) at *m/z* 935 [M- H]^-^, 773 [M- H- 162]^-^, 611 [M- H- 162× 2]^-^; IR ν_max_ (KBr) cm ^-1^: 3415 (OH), 2925 (CH), 1045; ^1^H-NMR (C_5_D_5_N) data see Table 1; ^13^C-NMR, see Table 2.

*Compound* ***3 (***(25*R*)-26-*O*-*β*-D- glucopyranosyl-5*β*-furostane-3*β*,12*α*,22,26-tetraol-3-*O*-*β*-D- glucopyranosyl(1→2)-*β*-D-galactopyranoside).White amorphous powder, m.p. 215–216 ºC, 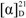 -28.7° (H_2_O, *c* 0.12). HR-ESIMS (positive mode) at *m/z* 1283.5891 [M+Na]^+^ (calcd 1283.5884). ESI-MS (positive mode) at *m/z* 1283 [M+ Na]^+^, 1121 [M+ Na- 162]^+^, 959 [M+ Na- 162× 2]^+^, 797 [M+ Na- 162× 3]^+^, 779 [M+ Na- 162× 3- H_2_O]^+^; ESIMS (negative mode) at *m/z* 1259 [M- H]^-^, 1097 [M- H- 162]^-^, 935 [M- H- 162× 2]^-^, 773 [M- H- 162× 3]^-^, 611 [M- H- 162× 4]^-^; IR ν_max_ (KBr) cm ^-1^: 3429 (OH), 2933 (CH), 1039; ^1^H-NMR (C_5_D_5_N) data see Table 1; ^13^C-NMR, see Table 2.

### 3.4. Acid hydrolysis of saponins

Each glycoside (5 mg) was heated in an ampoule with 15% aq. HCl (5 mL) at 110 ºC for 2 h. The aglycone was extracted with dichloromethane three times and the aqueous residue was evaporated under reduced pressure. Then, pyridine (1 mL) and NH_2_OH·HCl (2 mg) were added to the residue, and the mixture was heated at 100 ºC for 1 h. After cooling, Ac_2_O (0.5 mL) was added and the mixtures were heated at 100 ºC for 1 h. The reaction mixtures were evaporated under reduced pressure, and the resulting aldononitrile peracetates were analyzed by GC-MS using standard aldononitrile peracetates as reference samples. D-glucose and D-galactose were identified in a ratio of 4:1 for compound **1** and **2** and in a ratio of 2:1 for compound **3**. 

### 3.5. Platelets preparation

After obtaining consent, blood samples were collected from healthy volunteers (five men and five women; age range, 17–45 years) and 3.8% sodium citrate (nine parts blood, one part citrate) was added as an anticoagulant. All subjects avowed that they had not taken any anti-platelet drug at least 2 weeks prior to the extraction. Platelet-rich plasma (PRP) was prepared by centrifugation at 125×g for 10 min at room temperature. Platelet count was adjusted to 5 × 10^9 ^platelets/mL. PRP (400 μL) was added to the wells of a tissue-culture plate (24-well plate; well size 16 × 16 mm; Nunc) and pretreated with compounds (5, 20, 80, 320 μM) or AR-C67085MX (10 μM) at 37 ºC for 10 min, and then stimulated with 20 μM ADP for 5 min. 

### 3.6. Real-time reverse-transcriptase-polymerase chain reaction

The mRNA was collected from ADP induced active platelets following the manufacturers' recommended protocol. The mRNA was converted to cDNA and cDNA samples were kept at -20 ºC until needed. The CD40L and housekeeping gene *β*-actin primers for PCR analysis were based on the human platelet sequences and designed using the Primer 5 software. The forward and reverse primers were as follows:

CD40L mRNA: 5′ACATACAACCAAACTTCTCCC 3′ and 5′AGATGTTGTTTTACTGCTGGC 3′, (799 bp)

*β*-actin mRNA: 5’-AAGGATTCCTATGTGGGC-3’ and 5’-CATCTCTTGCTCGAAGTC-3, (362 bp)

The reaction mixture contained TaqMan universal PCR Master Mix (25 μL), 10 μM (2 μL) of each primer, TaqMan probe (2.5 μL) and cDNA (2 μL). It was adjusted to 22.5 μL with DNase/RNase free water. Conditions of the real-time-PCR were as follows: 50 ºC for 2 min, 95 ºC for 15 min, and for 40 cycles, 95 ºC for 15 s and 60 ºC for 1 min. The Ct value is defined as the number of fractional cycles in which the fluorescence emitted by the reaction mixture exceeds a preset threshold and marks the beginning of an exponential growth of the fluorescence signal. The results were analyzed with PE 5700 software.

### 3.7. Statistical analysis

All results were expressed as the mean±standard error (SE). Analyses were performed with SPSS software Version 13.0 (SPSS, Chicago, IL). ANOVA was used to determine if significant variation existed between group means. Two-side *p* values less than 0.05 were considered to be significantly.
